# Functional anomalies in healthy individuals with a first degree family history of major depressive disorder

**DOI:** 10.1186/2045-5380-2-1

**Published:** 2012-01-12

**Authors:** Francesco Amico, Angela Carballedo, Danuta Lisiecka, Andrew J Fagan, Gerard Boyle, Thomas Frodl

**Affiliations:** 1 Department of Psychiatry, Trinity College Dublin, College Green, Dublin 2, Ireland; 2 Institute of Neuroscience, Trinity College Dublin, College Green, Dublin 2, Ireland; 3 Adelaide and Meath Hospital incorporating the National Children's Hospital, Tallaght, Dublin 24, Dublin, Ireland; 4 St James's Hospital, Centre of Advanced Medical Imaging (CAMI), James's Street, Dublin 8, Dublin, Ireland

## Abstract

**Background:**

Individuals with major depressive disorder (MDD) process information with a bias towards negative stimuli. However, little is known on the link between vulnerability to MDD and brain functional anomalies associated with stimulus bias.

**Methods:**

A cohort of 38 subjects, of which 14 were patients with acute MDD and 24 were healthy controls (HC), were recruited and compared. The HC group included 10 healthy participants with a first degree family history of depression (FHP) and 14 volunteers with no family history of any psychiatric disease (FHN). Blood oxygen level dependence signals were acquired from functional magnetic resonance imaging (fMRI) during performance in a dot-probe task using fearful and neutral stimuli. Reaction times and the number of errors were also obtained.

**Results:**

Although MDD patients and HC showed no behavioral difference, the MDD group exhibited smaller activation in the left middle cingulum. The MDD group also showed smaller activation in the left insula when compared to the HC group or the FHN group. Finally, FHP participants exhibited higher activation in the right Heschl's gyrus compared to FHN participants.

**Conclusions:**

The present study shows that family risk for MDD is associated with increased activation in the Heschl's gyrus. Our results also suggest that acute MDD is linked to reduced activation in the insula and anterior cingulate cortex during processing of subliminal, not recognizable, masked fearful stimuli. Further research should confirm these results in a larger cohort of participants.

## Introduction

Most conceptions of the relationship between mood and emotions suggest that moods may potentiate matching emotional reactions (for example, irritable mood facilitates angry reactions [1]). Depressed individuals show more attention towards negative, anxiogenic stimuli [2] which has also been found to be a risk factor for developing major depressive disorder (MDD) [3]. Importantly, functional magnetic resonance imaging (fMRI) studies have demonstrated activation anomalies in both MDD patients and in patients at risk for depression during presentation of fearful images [4,5].

Interestingly, similar results have been found in healthy individuals with family history of depression (FHP) when compared to healthy individuals without any family history of the disease (FHN). FHP subjects exhibit impairment in emotion recognition [6] and have been shown to have higher amygdala and nucleus accumbens activation in response to the presentation of fearful faces when compared to age-matched FHN controls, in line with previous findings showing that FHP subjects have significantly elevated waking salivary cortisol when compared to FHN subjects [7]. However, when face viewing is accompanied by a constrained attention task (that is, having to rate nose width on the face and subjective fear while viewing the face), the differences between FHP and FHN subjects disappear whilst prefrontal activity increases [8]. This suggests that FHP subjects may be able to normalize emotion-related neural functions by focusing their attention and that face-viewing with unconstrained attention may leave room for aberrant psychological processes associated with the risk for developing MDD [8]. However, both behavioral and event related potential (ERP) studies have identified subtle deficits in selective attention among FHP individuals that may affect their ability to adequately regulate emotion under stressful circumstances [9].

In order to investigate the interplay between cognitive and emotion processing in both FHP and FHN participants, a masked emotion task combined with a cognitive task known to elicit cognitive processing bias in MDD might be useful to reveal performance differences between these two groups. In this context, neuropsychological studies in a task called 'dot-probe' suggest that depression is associated with an attentional bias towards negative information [10] and that effortful cognitive control of negative emotions can reduce the bias towards fearful stimuli [11]. Neuroimaging dot-probe studies suggest that unmasked fearful faces facilitate visual processing [12,13] and that the amygdala modulates fear responses in the occipital cortex [14]. Further, previous fMRI studies on participants performing in the dot-probe task (for example, [12]) have shown that the amygdala directs spatial attention to backward masked fearful faces through a network of brain structures that include the left anterior cingulate cortex (CC), right superior temporal sulcus and right lingual gyrus [15-17]. Other research in the dot-probe task has shown that individuals with MDD cannot avoid attending negative information in their environment [18] and FHP individuals attend selectively to sad faces [19]. Importantly, there is evidence that effortful control modulates the relationship between negative affectivity and attentional bias in the dot-probe task, with low levels of effortful control and high levels of negative affectivity predicting a preference for threat stimuli [11]. With respect to the fact that, when viewing subliminal masked stimuli, participants do not focus their attention on the masked stimuli, the dot-probe task is highly interesting because it might elicit activity associated with vulnerability to MDD [8]. However, to the best of our knowledge, little or no research has investigated putative functional anomalies in the brain showing during performance in this task in either MDD or FHP individuals.

In the present study, we hypothesized that patients with MDD compared to HC subjects, but also FHP subjects compared to FHN subjects, exhibit differences in emotional processing of fearful versus neutral stimuli when attention is biased during performance in a dot-probe task. Based on previous fMRI findings (for example, [17]) in a similar behavioral task [8,20-24], we selected the CC, amygdala, insula and prefrontal cortex as primary regions where we expected significant differences between groups to appear.

## Methods

### Participant recruitment

A cohort of 38 subjects aged between 18 and 65 was recruited. The healthy family history positive subjects (FHP, n = 10) were unaffected first-degree relatives of patients formally diagnosed with MDD according to the fourth edition of the Diagnostic and Statistical Manual of Mental Disorders (DSM-IV) and treated at the South-West Mental Health Services in Dublin, Ireland. However, the FHP subjects recruited were not the relatives of the MDD patients that participated in the study. Family history of MDD was assessed by a psychiatrist through a structured interview. Participants were asked whether any of their first degree relatives had been diagnosed with a psychiatric disease or had ever displayed symptoms of psychosis. Healthy volunteers without a history of psychiatric illness (FHN, n = 14) were recruited from the local community via announcements. The MDD group consisted of 14 patients with acute MDD attending our clinical outpatient services (Table 1). Of these, 4 were currently drug-free and came as new patients to our service, three received escitalopram, one fluoxetine, two venlafaxine, one venlafaxine plus mirtazapine, one sertraline plus mirtazapine, one sertraline, and one duloxetine plus mirtazapine.

**Table 1:** Demographic and clinical data of participants

	FHN (n = 14)		FHP (n = 10)		MDD (n = 14)				
							**Χ**^**2**^	**df**	***P***
Gender (female/male)	4/10		6/4		9/5		4.1	2/36	0.12
Handedness (right/left/ambidextrous)	14/0/0		8/2/0		12/1/1		5	4/34	0.3

	**Mean**	**SD**	**Mean**	**SD**	**Mean**	**SD**	**F**	**df**	***P***

Age (years)	35	9.4	33	10	41.2	10.3	2.2	2/36	ns
Weight (kg)	74	18	63.3	5	73.6	13	1.9	2/36	ns
Illness duration (years)	-	-	-	-	16	10	-	-	-
HDRS score	0.7	0.9	4.8	5.3	24.8	5	125	2/36	< 0.001^a^
Age of onset	**-**	**-**	**-**	**-**	25	11	-	-	-

	**Median**	**MR**	**Median**	**MR**	**Median**	**MR**	4.7	**df**	***P***

Alcohol intake per week (g)	16	16	60	26	28	18	4.6	2/36	ns
Cigarettes per day	0	15	1	23.5	0	21	5.7	2/36	ns

^a^There was a difference between the MDD patients and either the FHN (*P *< 0.001) or the FHP (*P *< 0.001) subjects in the HDRS; ^b^F-score from analysis of variance. df: degrees of freedom; MDD: patients with major depressive disorder; FHN: healthy participants without family history of MDD; FHP: healthy participants with family history of MDD; HDRS: Hamilton Depression Rating Scale for Depression; ns: not significant; SD: standard deviation, Χ^2^: Chi-squared score, MR: Mean Rank

For all subjects, a structured written observer interview and a structured interview carried out by two psychiatrists were used to assess demographic variables and medical history. Exclusion criteria were previous head injury with loss of consciousness, cortisol medication in their medical history, previous alcohol or substance abuse, co-morbidity with other mental illnesses, personality disorders, neurological or psychiatric disorder (Axis I or Axis II) or age over 65 years. No subject had ever received electroconvulsive therapy before investigation or took any psychotropic medications.

All participants included in the study filled out the following self- and observer-rated scales: the 21-item version of the Hamilton Depression Rating Scale for Depression [25], the Montgomery-Åsberg Depression Rating Scale [26], Beck's Depression Inventory [27] and the Structured Clinical Interviews for DSM-IV (SCID)-I [28] for psychiatric diseases and SCID-II [29] for personality assessment.

Handedness was determined by the Edinburgh Handedness Inventory [30]. Written informed consent was obtained from all subjects subsequent to a detailed description of the study. The study design, approved by the ethics committee of the Adelaide and Meath Hospital incorporating the National Children's Hospital and St. James' Hospitals, was prepared in accordance with the ethical standards laid down in the Declaration of Helsinki.

### Statistical analysis of clinical and demographic characteristics

Clinical and demographic data were analyzed using SPSS-16. Differences in gender and handedness were analyzed using Chi-square tests (see Table 1). Differences in age, weight and height were computed using a one-way analysis of variance (ANOVA). As alcohol intake (g/day) and the number of cigarettes smoked per day were found to be non-normally distributed, medians were calculated and a Kruskal-Wallis test was used to evaluate statistical differences between groups.

### Behavioral data

Behavioral measures analyzed included mean reaction time (RT) and the number of errors (that is, an error being made when the dot was indicated in the wrong side of the screen). Two conditions were compared: 'fear same' (dot and fearful face presented on the same side of the screen) and 'fear opposite' (dot and fearful face presented on opposite sides of the screen). There were a total of 19 'fear opposite' trials and 31 'fear same' trials for each participant. These trials were randomly selected by the presentation software. Unfortunately, due to a recording failure during the scanning sessions, some behavioral data were lost. Only the data that could be fully retrieved were included in the analysis (see Table 2). Both RTs and the number of errors for each condition were submitted to an ANOVA. A Bonferroni test was used for post hoc comparisons.

**Table 2:** Mean reaction times and total number of errors for conditions 'neutral' and 'fear'

	FHN (n = 10)		FHP (n = 7)		MDD (n = 5)				
	**Mean**	**SD**	**Mean**	**SD**	**Mean**	**SD**	**F**^**c**^	**df**	***P***
Neutral same^a^	4932	1076	4936	745	5342	606	0.402	2/20	0.67
Neutral opposite^b^	4903	1406	4818	697	5455	466	0.6	2/20	0.56

Fear same^a^	4589	721	4767	690	5582	684	4.4	2/20	0.05
Fear opposite^b^	5122	586	4734	710	5121	643.5	1.25	2/20	0.31
Fear (right+left)	4756	956	4728	700	5672	600	0.81	2/20	0.46
Neutral (right+left)	9835	2454	9754	1436	10797	1040	0.51	2/20	0.60
Number of errors	7	6.6	3.9	8	16.4	35	0.70	2/20	0.51

^a^Dot and fearful face presented on the same side of the screen; ^b^dot and fearful face presented in opposite sides of the screen; ^c ^F-score from analysis of variance. df: degrees of freedom; MDD: patients with major depressive disorder; FHN: healthy participants without family history of MDD; FHP healthy participants with family history of MDD; SD: standard deviation.

### fMRI data acquisition

Functional images were acquired on a 3-Tesla MRI scanner (Philips Achieva, The Netherlands). The MRI protocol consisted of the acquisition of a high resolution three-dimensional T1-weighted structural dataset [spoiled gradient recalled sequence with repetition time (TR)/echo time (TE) = 8.5/3.9 ms and 1 mm spatial resolution], followed by an fMRI experiment [spin-echo echo-planar imaging (SE-EPI) sequence with TR/TE = 2000/35 ms, in-plane resolution = 3 × 3 mm, 4.8 mm slice thickness, 304 dynamic scans each with 2 s duration].

Twenty five slices [dynamic scan time: 304, field of view: reference line: 230 mm, aperture: 230 mm, Fourier with a Hanning window (FH): 120 mm] covered the whole brain. Slices were positioned on the connecting line between the anterior and posterior commissure.

### Dot-probe task

Color mixed-race facial identities including 12 (6 male and 6 female) fearful and 12 (6 male and 6 female) neutral expressions [31] were randomly presented on a screen. A 7^th^ neutral female face from the same database was used as a mask. Each trial started with a fixation cross lasting between 1,000 and 2,500 ms. Next, a stimulus (randomly selected from neutral and fearful stimuli) was presented for 33 ms on the left or right visual field (LVF and RVF, respectively) and immediately masked by two neutral faces simultaneously presented (100 ms) on each visual field. Projections of masks were followed by a LVF or RVF target dot (750 ms) presentation with a jittered (500 ms to 2,000 ms) inter-trial interval. Subjects were required to respond as soon as possible by pressing a 'right' or 'left' button on a computer keyboard, according to the position of the target dot on the visual field. All participants were administered a practice trial. The total duration of the task was 10 minutes.

### fMRI data analysis

Standard preprocessing procedures were performed in SPM8 (Wellcome Trust Centre for Neuroimaging). The first six scans were not used to allow for T1 equilibration. The EPI images were then realigned to the first volume in order to correct for head movements. Realignment parameters were inspected visually to identify any potential subjects with head movement > 4.8 mm (slice thickness). Each participant's structural image was co-registered to the mean of the motion-corrected functional images using a 12-parameter affine transformation. Image slice time was corrected to TR/2. The structural images were segmented according to the standard procedure in SPM8 [32]. Spatial normalization to standard 3 mm × 3 mm × 3 mm Montreal Neurological Institute (MNI) space was then applied to functional images in order to allow for inter-subject analysis. Finally, these images were smoothed using an 8 mm full width at half maximum Gaussian kernel. Statistical parametric maps were calculated using a general linear model based on a voxel-by-voxel method [33].

First level single subject statistical parameter maps were created for each condition using the general linear model in SPM8. After parameter estimation, the following two contrasts were created: 'fear' > 'neutral' (F > N) and 'fear' < 'neutral' (F < N). Subsequently, these were entered into a full factorial second level analysis model using three groups (MDD, FHP and FHN) as factors. Age and gender were entered as cofactors. The statistical threshold was set to *P *< 0.05, with whole brain family-wise error (FWE) correction for multiple comparisons. Moreover, we reported differences with *P *< 0.001 in predefined regions of interest.

## Results

### Demographic data

The MDD group scored higher in the Hamilton Depression scale than either the FHN (*P *< 0.001) or FHP group (*P *< 0.001). No age, gender or handedness difference was found between groups (Table 1).

### Behavioral data

There was no significant difference between groups for either the RTs or the number of errors (Table 2).

### fMRI data (Table 3)

#### Contrast F > N

**Figure 1:**
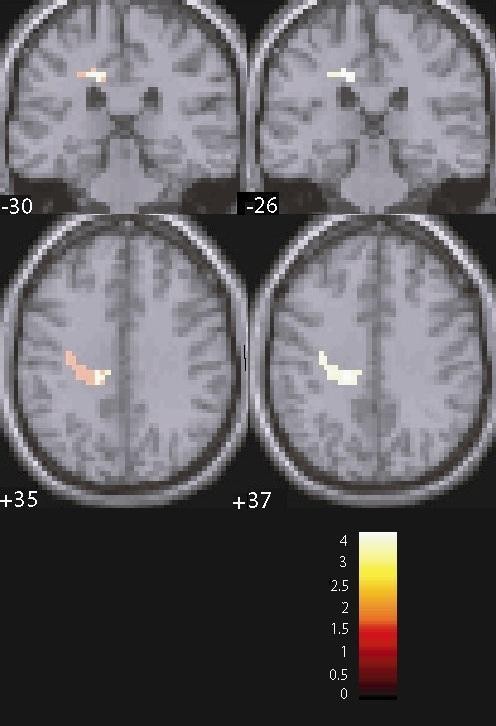
**Coronal and axial sections displaying activation differences between major depressive disorder (MDD) patients and healthy controls (HC) in the left middle cingulum (T = 3.82, *P*FWE = 0.041) and left insula (T = 4.19, *P *< 0.001, uncorrected)**. FWE: whole brain family-wise error correction.

**Figure 2:**
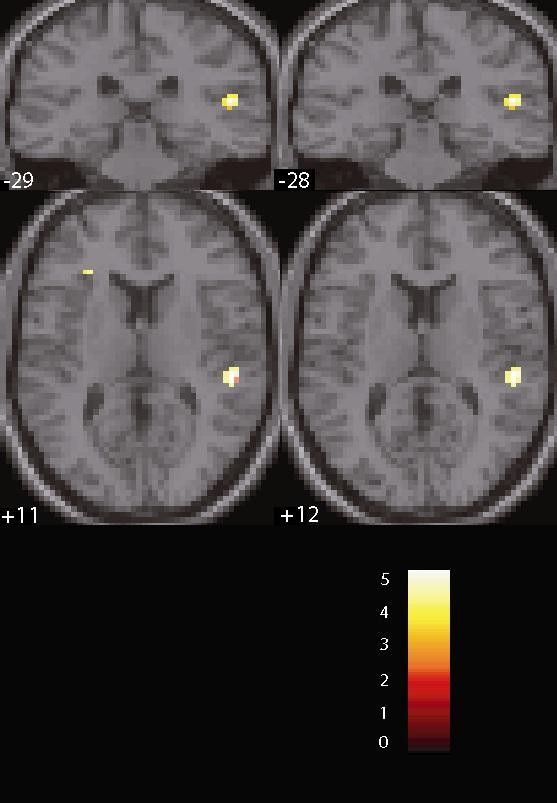
**Coronal and axial sections displaying activation differences between family history positive (FHP) and family history negative (FHN) participants in the right Heschl's gyrus (T = 4.60, *P*FWE = 0.01)**. FWE: whole brain family-wise error correction.

MDD patients exhibited smaller activation than healthy controls (HC) in the left middle cingulum (T = 3.82, *P *= 0.041, FWE corrected for multiple comparisons) and left insula (T = 4.19, *P *< 0.001, uncorrected), which also showed a trend for significance after correction for multiple comparisons (*P *= 0.072) (Figure 1). Smaller activation in the left insula was also found in the MDD group when compared to the FHN group (T = 4.43, *P *= 0.033, FWE corrected for multiple comparisons). Further, MDD patients had smaller activation in the left post-central gyrus when compared to FHN participants (T = 3.59, *P *< 0.001, uncorrected), although this difference did not survive FWE correction. Finally, the FHP group had greater activation in the right Heschl's gyrus when compared to the FHN group (T = 4.60, *P *= 0.018, FWE corrected for multiple comparisons) (Figure 2).

**Table 3:** Paired comparisons between healthy controls (HC, n = 24), family history negative healthy participants (FHN, n = 14), family history positive healthy participants (FHP, n = 10) and patients with major depressive disorder (MDD, n = 14)

Contrast	Comparison	Region	Region of interest *P *(FWE correction)	Ke	T	*P*uncorrected	x	y	z
F > N	MDD < HC	Left middle cingulum	0.041 (cluster corr.)	71	3.82	< 0.001	-15	-28	37
		Left insula	0.072	8	4.19	< 0.001	-27	29	7
	MDD < FHN	Left insula	< 0.033	11	4.43	< 0.001	-27	29	7
		Left post-central gyrus	ns	29	3.59	< 0.001	-27	-28	37
	MDD > FHN	-	ns	-	-	ns	-	-	-
	MDD < FHP	-	ns	-	-	ns	-	-	-
	MDD > FHP	-	ns	-	-	ns	-	-	-
	FHP > FHN	Right Heschl's gyrus	0.018	17	4.60	< 0.001	51	-28	13
	FHP < FHN	-	ns	-	-	ns	-	-	-
F < N	FHP < FHN	Right Heschl's gyrus	0.002	26	5.22	< 0.001	51	-28	13
	MDD < FHP	-	ns	-	-	ns	-	-	-
	MDD > FHP	-	ns	-	-	ns	-	-	-

F > N: "fear" > "neutral" contrast; F < N: "fear" < "neutral" contrast; FWE: family wise error correction; Ke: number of significant voxels; ns: not significant; T: t-value; x, y, z: coordinates in the Montreal Neurological Institute system.

#### Contrast F < N

The FHP group had smaller activation in the right Heschl's gyrus (T = 5.22, *P *= 0.002, FWE corrected for multiple comparisons) when compared to the FHN group.

## Discussion

While being presented with masked fearful stimuli, our participants showed significant differences in areas that are thought to play a key role in emotion processing, namely the CC and insula. Further, our results suggest a link between family history of MDD and functional anomalies in the Heschl's gyrus.

MDD patients showed reduced activity in the left middle CC when compared to the HC group, adding to previous findings suggesting an important role of CC anomalies in the diagnosis of anxiety disorders and/or depression [34]. In particular, this effect was observed when fearful facial expressions elicited stronger activation, in line with earlier fMRI research suggesting a role for the CC in orienting spatial attention to crude threat signals [8,17]. In this study, we found no effect in the amygdalae for all participants, suggesting a more direct involvement of the CC in attention recruitment during performance in the dot-probe task. This result might also agree with previous findings showing an involvement of the CC in shaping emotional expectancy in both healthy individuals and patients with MDD [35]. Interestingly, we found no effect in the prefrontal cortex, which contrasts with previous research showing prefrontal anomalies in both MDD and healthy participants with family history of MDD [8,24,36]. Our findings might suggest that our version of the dot-probe task is not sensitive to prefrontal activation anomalies in either MDD or FHP subjects, in line with previous research on the dot-probe task showing the involvement of the anterior CC, amygdalae, temporal and occipital cortices [12,13]. Although these results might need replication, an important consideration is to be made when comparing our data to previous findings in similar experimental contexts: while in the study of Monk *et al*. [8] participants were required to consciously shift their attention towards a specific feature of the stimulus presented (that is, participants were asked to rate the size of the nose in a given face), in the dot-probe task, stimulus perception is subliminal (emotional stimuli are masked by neutral stimuli). This might have an effect on how attention is recruited and might explain why, in our study, we detected no prefrontal effect. Previous research [15,17,37] has shown that the CC plays a key role in directing attention when an individual is not conscious of an emotional facial stimulus being presented. Further, recent ERP findings suggest that backward masked fearful face-elicited spatial attention facilitates behavior and modulates the early stage of facial processing [38].

Interestingly, when compared to the FHN group, the FHP group exhibited activation differences in the right Heschl's gyrus. In this area, FHP participants had greater activation for contrast F > N and smaller activation for contrast F < N. The Heschl's gyrus is a subregion of the superior temporal gyrus that, apart from being functionally involved in auditory processing, plays an important role in emotional processing, theory of mind and empathy [39,40]. Volumetric reductions in this area have been found in MDD patients, even after recovery from the disease [41]. Moreover, similar results have also been shown in bipolar disorder patients [42]. Our results implicate activation differences in superior temporoparietal areas between individuals with and without family history of MDD during exposure to fearful facial expressions. As only the right hemisphere was involved, our findings might also suggest a lateralization effect. This is perhaps in line with previous fMRI research suggesting a role of the right Heschl's gyrus during exposure to emotional (auditory) stimuli [43] and showing that the activation of auditory processing regions specialized for language, like the Heschl's gyrus, can be detected during performance in tasks requiring visual perception of the human face [44]. This might support the belief that this cortical area plays a role in acquired dynamic audiovisual integration mechanisms in the left superior temporal sulcus [44]. In this context, our results suggest a non-task specific role of the Heschl's gyrus in facial emotion processing, which is perhaps lost in MDD.

It is certainly interesting that MDD patients and FHP participants showed activation anomalies in different cortical areas, when compared to FHN participants. However, in the present study, these two groups consisted of unrelated individuals and whether MDD affects functional aberrances already detected before its onset in FHP subjects should be determined by future longitudinal studies.

The present study has a number of limitations. The subject sample was probably too small to reveal behavioral differences across groups. Additionally, the total number of HC participants was almost double than the number of MDD patients. This surely had an effect on our results. For example, our raw data suggested that MDD patients made considerably more errors than the HC group, although this could not be supported by statistical significance. Increasing the participant sample and having a comparable number of HC versus MDD participants would probably have yielded more definitive results. Further research in a larger sample of participants is also needed to confirm our RT analysis and comparisons (the RTs of some participants were lost due to a system failure).

Importantly, in the present study, we did not include images displaying faces conveying positive (happy) emotions. For this reason, we cannot rule out that our fMRI findings simply reflected brain activation associated with the presentation of emotional stimuli. In this regard, further fMRI research should aim at comparing brain activation relative to both happy and fearful faces. Participants were asked after scanning whether they could recognize subliminal images and confirmed that they did not detect them. Employing a detection task within the session would have been difficult, because participants already had to respond to the dots they saw after the shortly presented face images (100 ms). Further, future studies should also investigate correlations between behavioral and MRI data. Quite a substantial limitation of the present study is also represented by the inclusion of MDD patients with differences in medication which, as shown by previous fMRI research (for example, [23,45]), can affect brain activation. Finally, it also possible that the outcome of this research was affected by our recruitment method. We selected FHP participants as first-degree relatives of patients with well-known recurrent depression, but who did not necessarily take part in the study. As all the MDD patients recruited were assessed by the same psychiatrists, selecting relatives of MDD patients involved in the study might have contributed to ascertain family history of the disease in FHP participants. On the other hand, this would have introduced a genetic bias, whose selective effect on MRI data should be investigated in future research.

## Conclusions

Our results suggest that, in the dot-probe task, FHP subjects exhibit altered activity in the right Heschl's gyrus associated with subliminal presentation of fearful stimuli, indicating that lateralized alteration in the functionality of this cortical area could be associated with a higher risk of becoming depressed, although this should be confirmed by longitudinal studies on a larger population sample. Moreover, in individuals with MDD, the CC might mediate a preference for negative emotions as delivered by subliminally presented human faces. Further research is surely needed to explore the correlation between cortical and/or subcortical anomalies and behavioral responses in a similar experimental setting and to investigate putative therapeutic effects of psycho- and pharmacotherapy on the activation anomalies we detected.

## Abbreviations

ANOVA: analysis of variance; CC: cingulate cortex; DSM-IV: Diagnostic and Statistical Manual of Mental Disorders; SE-EPI: spin-echo echo-planar imaging; ERP: event related potential; FHN: family history negative; FHP: family history positive; fMRI: functional magnetic resonance imaging; FWE: family-wise error; HC: healthy control; LVF: left visual field; MDD: major depressive disorder; RVF: right visual field; RT: reaction time; TE: echo time; TR: repetition time.

## Competing interests

The authors declare that they have no competing interests.

## Authors' contributions

TF, AC and DL acquired MRI data. AF and GB supervised MRI data acquisition. FA and TF carried out data analysis and wrote the present manuscript. All authors read and approved the final manuscript.
